# Selection of a Very Active Microbial Community for the Coupled Treatment of Tetramethylammonium Hydroxide and Photoresist in Aqueous Solutions

**DOI:** 10.3390/ijerph15010041

**Published:** 2017-12-27

**Authors:** Giulio Moretti, Federica Matteucci, Matteo Saraullo, Francesco Vegliò, Maddalena del Gallo

**Affiliations:** 1 Department of Life, Health, and Environmental Sciences, University of L’Aquila, 67100 L’Aquila, Italy; giulio.moretti1@graduate.univaq.it (G.M.); federica.matteucci@univaq.it (F.M.); 2 Department of Industrial and Information Engineering and Economics, University of L’Aquila, 67100 L’Aquila, Italy; matteo.saraullo@hotmail.it (M.S.); francesco.veglio@univaq.it (F.V.)

**Keywords:** Tetramethylammonium hydroxide, photoresist, DGGE, NGS, microbial ecological indices

## Abstract

Aerobic treatment of wastewater containing Tetramethylammonium hydroxide (TMAH) and photoresist was investigated using a lab scale reactor inoculated with activated sludge coming from urban wastewater treatment that never received TMAH before. The consumption of TMAH was monitored by liquid ion chromatography. Biodiversity indices were calculated from Denaturing Gradient Gel Electrophoresis (DGGE) bands distribution and used to estimate changes in community composition related to adaptation to the new feeding compound. The first week of adaptation was crucial, and it was analyzed in detail: many organisms died, and the microbial community suffered a great shock. TMAH levels remained constant through the first four days, and then suddenly dropped to undetectable, and at the same time NH_4_^+^ increased. When the community showed complete adaptation, predominant groups of bacteria were obtained by the Illumina sequencing of 16s rDNA amplicons, to provide insights on ecology of the adapted community, focusing on the main actors of TMAH abatement. Richness of species (*Rr*) peaks suggest that the development of TMAH-consuming bacteria leads to persistent consortia that maintain toxicity resistance over time. This showed adaptation and changes of the population to the different feeding conditions, and it opens new perspectives in the in situ treatment of these important residues of industrial processes without relying on external processing plants.

## 1. Introduction

Tetramethylammonium hydroxide (TMAH) is a quaternary ammonium salt widely used in electronic industry in the production of semiconductors, in combination with a UV-sensible solvents mix known as photoresist. TMAH use in photolithography is due to its organic alkali properties and phase transfer being soluble in both water and methanol [1].

This process produces an effluent that, due to its toxicity [2], needs to be treated before being discharged. TMAH is classified as hazardous for humans and dangerous for most living organisms, including fungi and bacteria [3].

In the literature, many different treatment processes have been described, including chemical, physical, biological, and combined ones.

Lei et al. [4] studied a biological process to treat wastewater containing TMAH, dimethyl sulfoxide (DMSO), and mono-ethanolamine (MEA) in aerobic and anaerobic conditions. In both cases, degradation of TMAH was possible, but in anaerobic studies, an inhibitory effect was recorded with a high concentration of this salt. Otherwise, in the aerobic condition NO_x_ are produced.

Asakawa et al. [5] proved that some Methanosarcinaceae could use TMAH as carbon source to produce CH_4_. Chang et al. [6] produced a study with similar results using an upflow anaerobic sludge blanket (UASB) reactor. The degradation of TMAH reached 95%, the concentration of 10 g/L was not toxic to Archaea, and the process produced a gas flow of CH_4_ and CO_2_, but NH_3_ remained in the liquid phase. Lin et al. [2] studied a two-stage ANAMMOX process, obtaining a conversion to nitrogen of 90%. In addition, chemical oxidation of TMAH is possible. With this kind of treatment, the main problem is related to NO_x_, N_2_O, and CO that are produced during the process. Hirano et al. [7] demonstrated that, combining the pyrolysis of TMAH with its catalytic oxidation is possible to treat this ammonium salt obtaining non-poisonous gas composed of H_2_O, CO_2_, and N_2_.

TMAH adsorption is also a possible option for treating this wastewater. Prahas et al. [8], using various activated carbon (AC), obtained a maximum adsorption capacity of 27.7 mg/g AC, and they have described the equilibrium using Langmuire isotherm. Nishihama et al. [9] used zeolites and they have measured equilibrium concentration on solid phase of 1.13 · 10^−2^ mmol/g. Kelleher et al. [10] obtained an equilibrium concentration on solid phase of 270 mg/g using mesoporous silicate material.

Citraningrum et al. [11] studied TMAH removal from aqueous solution using different cationic ion exchange resins. With strongly acidic cation exchange resins (SAC), removal of 177.55 mg/g of TMAH was obtained in 20 min, with weakly acidic cation exchange resins 186.99 mg/g removal reached in 40 min.

Instead, Den et al. [12], by combining photochemical oxidation with biological process, treated wastewater containing TMAH and photoresist, and obtained an efficiency of total organic carbon (TOC) reduction of about 97% for TMAH.

Biodegradation is possible, but is potentially a cost-effective way of treating TMAH contaminated waters. In a microbial system, a substrate assumes the habitat function, and the conditions under which it can be metabolized assume the functions of the ecological niche. In a preliminary experiment, we tried to isolate a single species that was able to grow and to presumably metabolize TMAH. However, the results were marginal: it is very difficult to isolate single microorganisms that are able to utilize tetramethylammonium, confirming that this it is minimally biodegradable, or, in technical terms, recalcitrant. Quaternary ammonium salts are known antimicrobials [13] and their effect is due to the interactions of the positive quaternary ammonium charge with groups negatively charged on biological membranes. In particular, tetramethylammonium is a very stable organic base. Poor TMAH removal from wastewaters is due to the null reactivity of the quaternary ammonium ion towards electrophiles, oxidants, acids, and most nucleophiles. Furthermore, absence of a N^+^-C-C-H bond in TMA group prevents it to undergo quaternary ammonium reactions such as Sommelet–Hauser rearrangement, Stevens rearrangement, Hofmann elimination, and Emde degradation. Pathway of tetramethylammonium biological degradation is still unclear.

In the present study, treatment of wastewater containing TMAH and photoresist was investigated using a lab scale reactor supplied with activated sludge coming from a urban wastewater plant. Objective of this study was to check the feasibility to treat, biologically, this kind of aqueous residue by adapting a common wastewater microbial community. Changes in response to the presence of TMAH in the microbial community structure was monitored by Denaturing Gradient Gel Electrophoresis (DGGE). When community structure stabilized, its members were identified by New Generation Sequencing (NGS, Illumina MySeq). This provides new insights into adaptation and change of the population to the different feeding conditions, and it opens new and concrete perspectives in the treatment of these important residues of industrial processes. We demonstrate that it is possible—with simple procedures—to treat these industrial effluents directly in situ, without relying on external processing plans.

## 2. Materials and Methods

### 2.1. Reactor Set Up and Batches

As a model to study the biological oxidative TMAH removal into domestic wastewaters, a 5 L lab scale reactor, model BIOSTAT B, equipped with a thermometer, an oximeter, and an electric engine stirrer on the top, was operated for one month. Inside the reactor, a half-moon sparger connected to a 20 nm filter allowed the air to bubble inside. All parameters were monitored from a controlling tower connected with the reactor. 

The wastewater containing TMAH and the exhausted photoresist were furnished by LFoundry—a company with operating headquarter in Avezzano (L’Aquila, Italy)—stocked in plastic tanks. Tetramethylammonium and ammonium ion concentrations in native solution and in samples were measured with ICS-1000 (Dionex, Sunnyvale, CA, USA) using a Thermo Scientific AS-DV autosampler (Waltham, MA, USA). All samples were centrifuged and filtered with a 0.2 μm syringe filter PURADISC™ 25 NYL. The Dionex IC was equipped with an IonPacCG-18 column as the guard and cation analytical column, without suppressor, and a conductivity detector. Analytes were detected using a DS6 heated conductivity cell thermostated at 30 °C. The eluent conductance was recorded at Hz, and the chromatographic peaks were analyzed by Cromeleon 7 software package (Thermo Fisher Scientific, Waltham, MA, USA). 

The activated sludge was sampled from the domestic water treatment plant of the same company (not containing neither TMAH nor photoresist) in wide neck plastic bottles. They were kept under a fume hood and left open, in contact with atmospheric air, for 14 days to allow the complete oxidation of residual organic matter inside.

A mineral culture medium, supplemented with a small amount of yeast extract, was prepared according the composition shown in Table 1. Yeast extract final concentration in the reactor was 250 μg/L.

Carbon content in the feeding was increased to balance the carbon/nitrogen ratio to 20/1 by adding another industrial residue known as photoresist, consisting of a mixture of organic compounds with a high Total Organic Carbon (TOC) value (615 mg/g), photoresist is heat sensitive and could not be steam sterilized.

Before use, the TMAH wastewater was neutralized to pH 7 by 1 M H_2_SO_4_. The feeding for the reactor was obtained by mixing 1.93 L of TMAH residue, 20 mL of photoresist, and 50 mL of culture mineral medium. The experimental reactor was kept at room temperature (20 ± 2 °C), under 70 rpm stirring, at air-oxygen saturation. Each day 120 mL of solution was sampled with a peristaltic pump and stored frozen until used for analysis.

**Figure 1:**
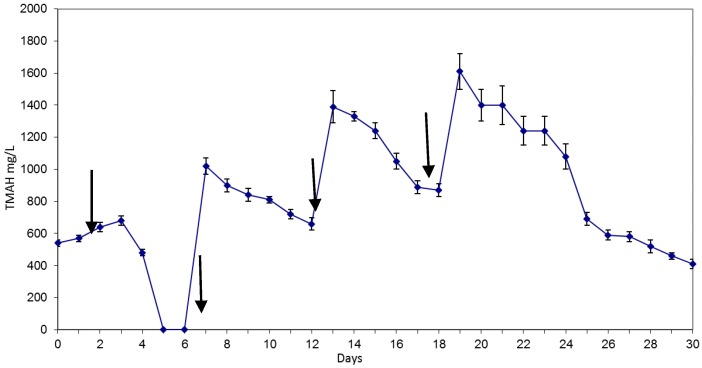
Tetramethylammonium concentration in the reactor at different intervals of time sampling. Reactor was loaded with fresh medium containing TMAH on days 0, 7, 13, 19. Arrows indicate TMAH and Photoresist medium addition.

Biomass enrichment was obtained through a series of consecutive batches, in which one litre of sludge was kept in the reactor and fed with fresh medium as follows:

- Batch T: the reactor was filled with 2 L inoculum and 2 L of feeding solution.
- Batch R (days 7 to 12), batch A (days 13 to 18), batch M (days 19 to 24): at the beginning of each batch, 1 L of activated sludge coming from the previous batch was kept in the reactor and fed with 2 L of feeding solution. Each batch was 6 days long.
- Batch M0 (since day 25): in this batch, 1 L of activated sludge coming from batch M was fed with 2 L of distilled water and 50 mL of mineral culture medium. Batch’s timeline and TMAH concentration at the different times are outlined in Figure 1.

### 2.2. Biological Analyses

Colony counts were done on both Luria-Bertani rich medium and AGAR-TMAH selective medium composed as follows: TMA-SO_4_ solution 500 mL/L (TMA final concentration 1 g/L), 1.5% Bacteriological Agar; 1 mL/L Standard microbial vitamin solution (ATCC MD^®^-VS, Manassas, VA, USA), 1 mL/L Hoagland trace element solution. 

Lowry’s protein assay was performed on lysate cells to estimate biomass production. Bacterial DNA from samples was extracted with a Soil DNA Isolation Kit (Norgen, Thorold, ON, Canada); 16s rDNA v-3 region was amplified by PCR, using Bacterial universal primers 341F (5′-CCTACGGGAGGCAGCAG-3′) with GCclamp and 518R (5′-ATTACCGCGGCTGCT-3′), referring to the standard amplification protocol [14]. Amplified 16s rDNA regions were purified with a DNA purification kit (Zymo Research, Irvine, CA, USA), quantified with Nanodrop 2000, and diluted with PCR water (Milli-Q, Merk KGaA, Darmstadt, Germany). Denaturing Gradient Gel Electrophoresis (DGGE) was performed on 8% polyacrylamide gel in 1× TAE buffer with a denaturant gradients 40% to 65% (urea and formamide) in DCode apparatus (Bio-Rad, Hercules, CA, USA), run overnight (for 18 h) at a constant voltage of 60 V and at temperature of 60 °C. DGGE was repeated three times to have a statistically significant data set that was used to estimate biodiversity indices for each time point sampled. Biodiversity indices were calculated as follows:

Range-weighted Richness: $Rr\;=\;M^{2}\;\cdot \;\Delta g$;
Functional Organization: *Fo* = y value for x = 20% on Pareto Lorentz distribution of species; Simpson Diversity Index: 1 − D = 1−Σ(n/N)^2^;
Simpson Evenness Index: $Ed\;=\;\frac{1\;-\;D}{\left(1-1/M\right)}$;

where *M* = number of species (bands in a lane); ∆*g* = normalized gradient distance between first and last band; *n* = intensity of a band; *N* = sum of band intensities on a lane. 

Once the community was stabilized as number of bands and biodiversity indices, whole community 16s DNA sequencing was performed, in double, to have a significant insight on the fastest species able to grow at high concentration of TMA-SO_4_ wastewaters. DNA sequencing has been performed with Illumina MySeq on 16s rDNA genes amplified with bacterial primers 27F (5′-AGAGTTTGATCCTGGCTCAG-3′) and Uni1492R (5′-GGTTACCTTGTTACGACTT-3′) at GATC Biotech (Konstanz, Germany).

## 3. Results and Discussion

### 3.1. Reactor Monitoring, Early Phase

Batches timeline and TMAH concentration all along the experiment are outlined in Figure 1. 

Microscopy observation of colonies grown on TMAH as sole carbon source showed that cells were small cocci or rods, and Gram-negative bacteria. 

**Figure 2:**
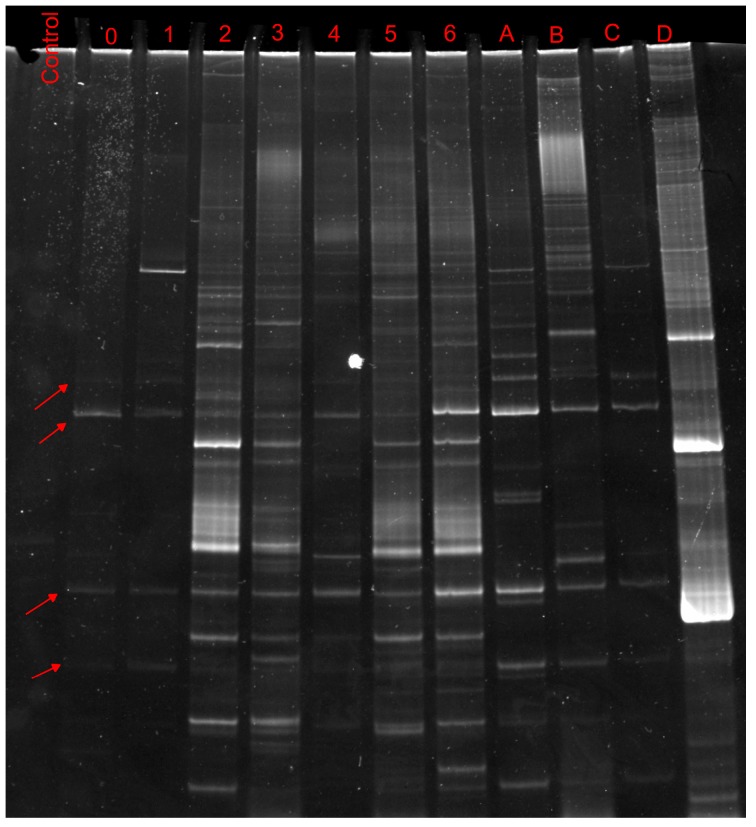
DGGE analysis, 45–65% of the first week of adaptation (days 0–6) compared with amplicons from selected colonies on plates (A–D). Biodiversity was unstable, but is possible to see how bands from organism C are present in all the phases.

A DGGE gel obtained from samples T0 to T6 (first batch), monitoring the first week of community adaptation, is shown in Figure 2. Two series of bands were present consistently at every time and were also present in TMAH-consuming consortia isolated trough streaking method on plates with TMAH as sole carbon source (A to D). This suggests that these organisms played a vital role in community development on toxic substances. 

**Figure 3:**
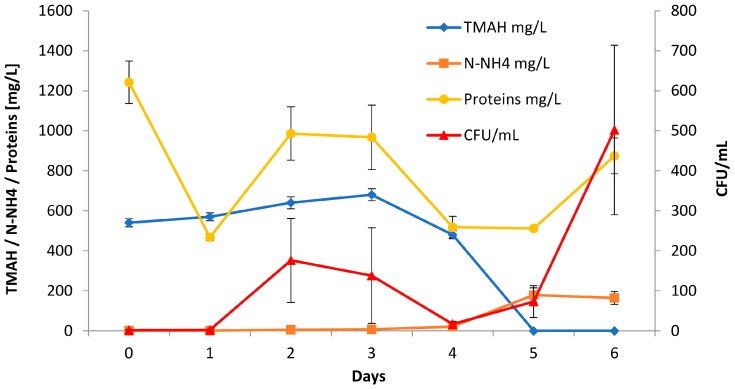
Parameters monitoring the first batch of bioreactor. TMAH degradation started after 4 days (♦); meanwhile, NH_4_^+^ was accumulated in the medium (■). Two different phases of growth are visible in CFU/ml counts (▲) and in Protein Lowry assay obtained on lysed cells (●). In the first two days, there was no visible growth on plates and the protein content dropped significantly.

In the first two days of adaptation, the community suffered a great shock. TMAH levels remained constant through the first 4 days and then suddenly dropped to undetectable when NH_4_^+^ increased (Figure 3). In the following three batches, the rate of degradation remains fairly constant: 70 mg/L per day in the second week, 100 mg/L per day in the third and fourth, with a reduction of about 15% per day. The amount of TMAH present in the culture medium, however, affects the degradation rate: in the first batch the amount is rather low, less than 600 mg/L, and the degradation rate is high. In subsequent batches the degradation rate remains constant, but the amount of TMAH increases for continued additions.

Figure 3 shows protein content and Colony Forming Units (CFU) count in relation to TMAH abatement in the medium. We observe a typical growth curve in the first phase, with a death phase that peak in T4, in which the community consumed the easiest nutrients in the medium, namely photoresist components; then, a second growth of microorganism consuming the TMAH was completely established.

**Figure 4:**
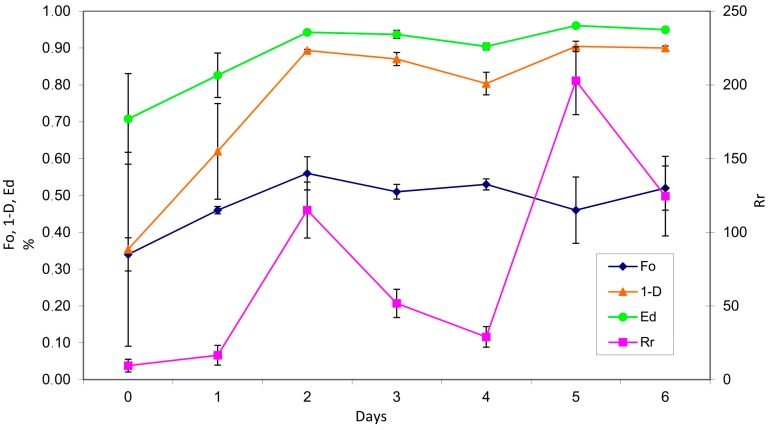
Species distribution (1 − *D*, ▲), Species Evenness (*Ed*, ●), Functional Organization (*Fo*, ♦), and Range-weighted Richness (*Rr*, ■) of bacterial community over time, obtained by DGGE. T0, T1: the microbial community suffered a shock, and most species were undetectable; T2, T3: community species distribution stabilized at optimal values; T4: TMAH resistant bacteria prevailed in the medium, while other species were undetectable; T5, T6: microbial community restored at optimal values, with consistent production of biomass.

*Fo* index shows that the community quickly organized itself at the beginning and then loosened its organization due to cell death when resources became limiting; finally, it organized again when TMAH was completely utilized. Biodiversity distribution (1 − *D*) and Evenness (*Ed*) Simpson’s indices raised slowly during the first 48 h and then stabilized, with a fluctuation in T4, in which TMAH abatement was maximal and metabolically active bacteria were predominant (Figure 4). This indicates that, as suggested by Torresi et al. [15], that degradation is positively influenced by community richness and evenness.

If we compare the phase of adaptation and the late phase, it is possible to notice that Richness, Diversity, and Evenness rose considerably among samples. Especially for Diversity and Evenness indices, standard deviation between samples was tenfold lower, demonstrating that the community composition was very stable over time. 

**Figure 5:**
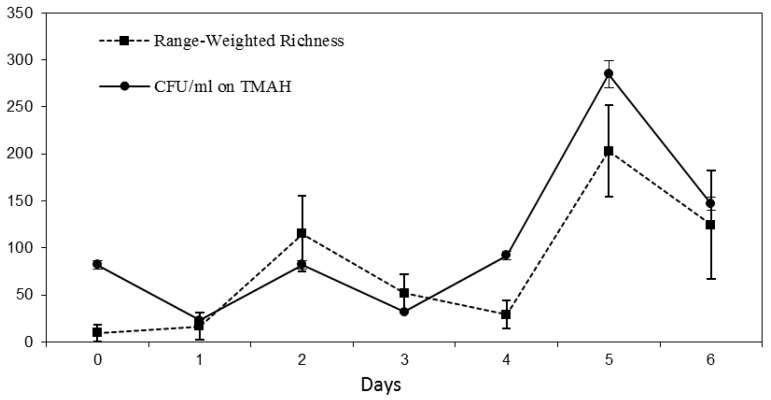
Richness of species into the reactor obtained by DGGE in comparison with Colony Forming Unit on Agar media with TMAH as sole carbon source.

*Fo* is an index of response to environmental changes in the community: it raised twice in the ecological succession from contaminated to clear waters. Richness of species (*Rr*) peaks were comparable with CFU counts on TMAH selective medium, suggesting that the development of TMAH consuming bacteria leads to persistent consortia with cooperative, not competitive, behaviour (Figure 5). 

Those results confirm that biodiversity in the reactor was related to TMAH abatement and, therefore, a high number of species that feed on TMAH were established within a week.

### 3.2. Reactor Monitoring, Late Phase

**Figure 6:**
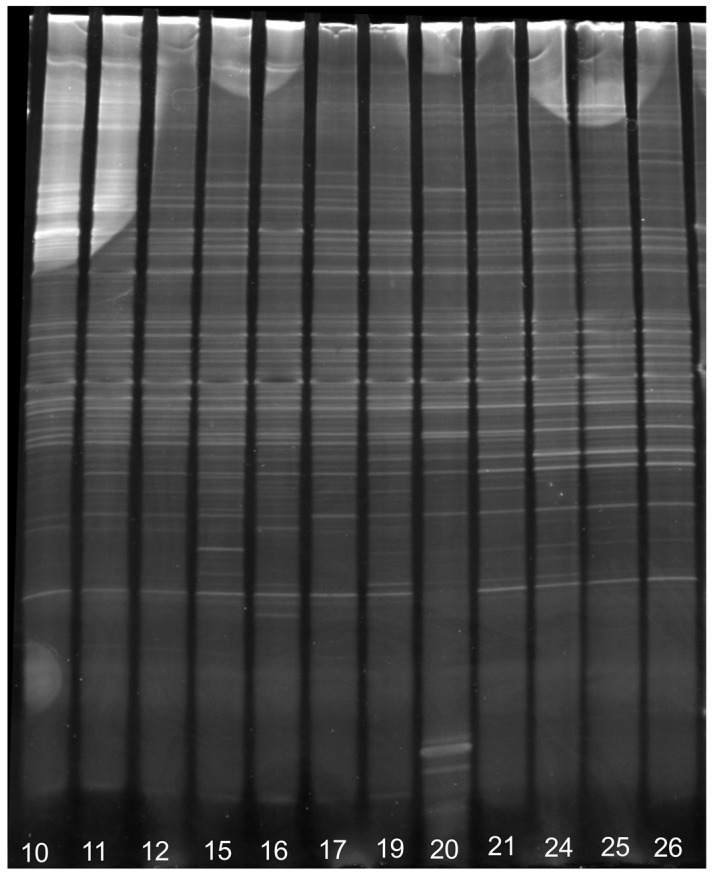
45–65% DGGE analysis on selected samples ranging from days 10 to 26 shows a constant community over time. The changes are almost imperceptible, and the composition of the community remains almost unchanged until the end of the experiment, indicating that from the original inoculum the best possible community for this function was selected.

After one month of continuous feeding with TMAH solution, the community stabilized with barely no changes in band numbers and position (Figure 6). 

**Figure 7:**
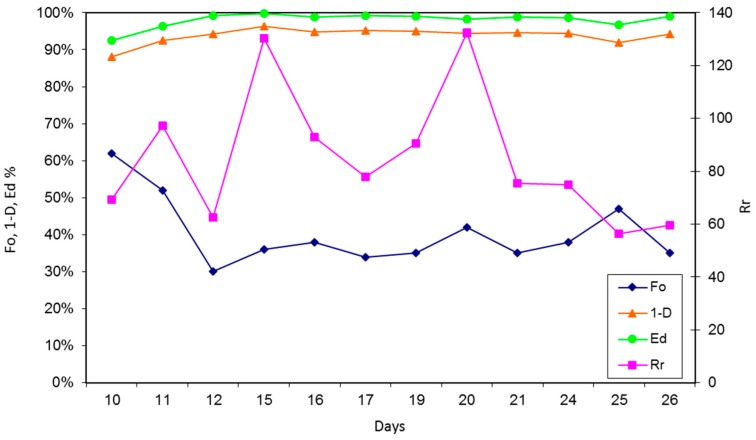
Species distribution (1 *− D*, ▲), Species Evenness (*Ed*, ●), Functional Organization (*Fo*, ♦), and Range-weighted Richness (*Rr*, ■) of bacterial community over time, obtained by DGGE (days 10 to 26). Despite punctual variations of specie numbers, at the end microbial community was consistently evenly distributed and organized.

If we compare the biodiversity indices obtained from the second part of the experiment (days 15–30) with those of the first part (days 0–6), we note that *Fo* decreases in value and stabilizes at around 30–45%, indicating a more uniform and adapted community. When the photoresist solution was added to the medium, due to the high TOC of this product, Ranged Richness of species increased every time that fresh medium was added to the reactor but, after having utilized all photoresist, only the species utilizing TMAH grew, and community structure restored within a few days. Despite those peaks, richness of species decreased sensibly in value and set around 60. Simpson’s indices of species diversity (1 − *D*) and evenness of distribution (*Ed*) increased in value, respectively, by 7% and 5%, and stayed constant for a long period of time (Figure 7).

The decrease of species richness and organization, and the increase of diversity and evenness index, means that, driven by TMAH selective force, a highly adapted community was selected from the initial one. At the same time, TMAH removal efficiency reached a maximum value of 160 mg/L per day when TMAH concentration was between 1 and 1.4 g/L.

To understand roles and properties of different members in the community, samples taken from the last two days (25 and 26) were selected for Next Generation Sequencing (Table 2). These samples were chosen because they had identical DGGE patterns and were at least one week away from the last feeding. Community composition obtained through Illumina MySeq confirmed that most of the bacteria selected by the presence of TMAH were Gram-negative, belonging to families able to grow on a large number of substrates, such as Proteobacteria, with a wide representation of Gammaproteobacteria. 

Main genus representation in sequence reads were *Comamonas* (18.2 ± 5.7%), *Pseudomonas* (18.5 ± 5.3%), *Stenotrophomonas* (12.3 ± 3.1%), and *Brevundimonas* (11.4 ± 3.8%), while others sequence reads were spread in various groups belonging to families *Sphingomonadaceae* (8.1 ± 0.9%), *Sphingobacteriaceae* (4.05 ± 2.1%), *Methylophilaceae* (4.05 ± 3.0%), *Xanthobacteraceae* (3.3 ± 0.1%), and the genus *Rhodococcus* (1.9 ± 0.5%) (Table 3). The bacterial population consisted predominantly of high adaptable bacteria that possess the ability to grow on solvents, methylamines, and methanol, and most are denitrifiers. Unique features of these Gram-negative bacteria are the ability to reduce nitrous oxides generated by the oxidation of TMAH, and most of them are useful for bioremediation of pollution from chlorinated compounds (*Pseudomonas knackmussii* B13, [16], *Xantobacter autotrophicus* [17], and aromatic compounds, such as *Comamonas* spp. [18]; *Stenotrphomonas* spp. [19]). Instead, the presence of *Sphingomonas* and *Sphingobacteria* may be due to the sphingolipids present in their membrane [20].

## 4. Conclusions

The bibliography remains exhaustive regarding the possibility and the need to treat these particular wastewaters, and it indicates various methods for monitoring the microbial community but does not go further: it remained a vacuum to be filled in the dynamics of the process of degradation of the TMAH and the conditions in which it occurs.

Our results show that the community adapted quickly to the change of nutrients and stabilized over a new equilibrium state within a week when in constant presence of Tetramethylammonium, and any successive perturbation did not change the community structure. This means that a normal wastewater treatment plant, taking into account particular parameters such as pH, micronutrients, C/N ratio, and nutrient and oxygen feeding, can be converted into TMAH wastewaters treatment without noticeable negative effects. 

Therefore, the results obtained in this experiment can be utilized for a biodegradation process that involves lower costs compared to traditional methods. The identification and selection of a highly active microbial community can immediately provide industry with a rapid process of degradation of these highly polluting quaternary ammonium compounds. Knowing the composition of the microbial community, it is also possible to prepare an inoculum to be used in waste-water treatment plants. 

These results are also interesting from the Microbial Ecology point of view: ecological indices allow us to fully monitor the structure and organization of the entire bacterial community without the need to identify the individual species. The results obtained showed a much more organized and stable community over time, even in the long run, indicating that it is specialized and, given the starting community, the best community for the desired function was selected.

Our knowledge is not sufficient to understand all the mechanisms that take place in biological sludge for the purification of these waters; however, the concepts expressed in the present work can provide a method of investigation useful for the selection of a microbial community and the development of conditions for the degradation of pollutants of anthropogenic origin.

Adapting to the change of one or more environmental parameters is one of the key mechanisms for life preservation. Microorganisms live in very complex communities, and thanks to their rapid metabolism and reproductive speed, they allow us to observe the adaptation of a community in a short time. 

However, we demonstrated that the complete transformation of Tetramethylammonium in its by-products happens through a syntrophy of various microorganisms. A selection of an unusual microbial community that has never been studied before was achieved, which definitely deserves more thorough analysis.

## Figures and Tables

**Table 1:** Culture medium composition.

Culture Medium Composition	
Component	g/L
CuCl_2_	0.05
Na_2_MoO_4_	0.05
NaHCO_3_	0.7
K_2_HPO_4_	0.1
MgSO_4_	0.1
FeCl_3_	0.1
Yeast extract	0.01

**Table 2:** Main genera of bacteria, based on percentage of reads, with representative OTU for every genus (if applicable), and main features of the representative OTU.

Genus/Family	G1	G2	Average	Error	Representative OTU	Features	References
*Comamonas*	22.3	14.2	18.25	5.728	*Comamonas granuli*	grows on solvents	[18]
					*Comamonas nitrativorans*	Denitrifier	
*Pseudomonas*	14.3	21.8	18.05	5.303	*Pseudomonas knackmussii* strain B13	grows on polyamines	[16]
					*Pseudomonas nitritireducens*	can reduce nitrate	[21]
*Stenotrophomonas*	14.5	10.1	12.3	3.111	*Stenotrophomonas humii*	can reduce nitrate/nitrite	[19]
*Brevundimonas*	14.1	8.7	11.4	3.818	*Brevimonas abyssalis/B. viscosa*	nitrate reduction—grow on tween	[22]
*Sphingomonadaceae*	8.8	7.4	8.1	0.990	N.A		
*Sphingobacteriaceae*	2.5	5.6	4.05	2.192	N.A		
*Methylophilaceae*	1.8	6.3	4.05	3.182	*Methylobacillus glycogenes*	grows on methanol and methylamines	
*Xanthobacteraceae*	3.4	3.2	3.3	0.141	*Methylobrevis pamukkalensis*	grows on methanol and methylamines	[23]
*Xanthobacter*	2.5	2.6	2.55	0.071	*Xantobacter autotrophicus*	Methilotroph, Bioremediation of clorinated compounds	[17]
*Rhodococcus*	1.5	2.3	1.9	0.566	N.A		

**Table 3:** Biodiversity indices average and standard deviation among samples.

	*Fo*	*Rr*	Diversity	Evenness
Average (adaptation)	0.48	34.8796	0.76365	0.88993
Average (developed community)	0.40	84.9199	0.93809	0.98028
Std. Deviation (adaptation)	0.072736	30.8343	0.20693	0.09237
Std. Dev. (developed community)	0.091187	25.2664	0.02128	0.01995
